# Hybrid microscaffold-based 3D bioprinting of multi-cellular constructs with high compressive strength: A new biofabrication strategy

**DOI:** 10.1038/srep39140

**Published:** 2016-12-14

**Authors:** Yu Jun Tan, Xipeng Tan, Wai Yee Yeong, Shu Beng Tor

**Affiliations:** 1 Singapore Centre for 3D Printing, School of Mechanical & Aerospace Engineering, Nanyang Technological University, 50 Nanyang Avenue, 639798 Singapore

## Abstract

A hybrid 3D bioprinting approach using porous microscaffolds and extrusion-based printing method is presented. Bioink constitutes of cell-laden poly(D,L-lactic-co-glycolic acid) (PLGA) porous microspheres with thin encapsulation of agarose-collagen composite hydrogel (AC hydrogel). Highly porous microspheres enable cells to adhere and proliferate before printing. Meanwhile, AC hydrogel allows a smooth delivery of cell-laden microspheres (CLMs), with immediate gelation of construct upon printing on cold build platform. Collagen fibrils were formed in the AC hydrogel during culture at body temperature, improving the cell affinity and spreading compared to pure agarose hydrogel. Cells were proven to proliferate in the bioink and the bioprinted construct. High cell viability up to 14 days was observed. The compressive strength of the bioink is more than 100 times superior to those of pure AC hydrogel. A potential alternative in tissue engineering of tissue replacements and biological models is made possible by combining the advantages of the conventional solid scaffolds with the new 3D bioprinting technology.

Three-dimensional (3D) printing is inspiring innovation in many areas, particularly in the 3D printing of biomaterials^1^,^2^. 3D printing of scaffolds^3^,^4^,^5^ have been demonstrated by using bio-inert materials of metals^6^, ceramics^7^, polymers^8^, hydrogels^9^ and even smart materials^10^. 3D bioprinting is the layer-by-layer spatial patterning and assembling of living cells together with biologics and/or biomaterials with a prescribed organization, forming a 3D living cellular construct^2^,^3^,^11^. It is therefore highly challenging as living cells have to be delivered in each bioprinted layers without drastically affecting the cells phenotype and viability. At the same time, the biological constructs have to be self-supported without collapsing. Currently, common modus operandi reported in literatures are the 3D bioprinting using bioinks of the cell-laden hydrogels^12^,^13^ or the high cell density tissue spheroids as well as tissue strands^14^,^15^. Here we propose a 3D bioprinting strategy introducing the conventional scaffold-based tissue engineering (TE) approach.

It was thought that the solid scaffold-based TE methods and the solid scaffold-free bioprinting approaches cannot be integrated^5^,^16^. The solid scaffold-free cell-laden hydrogel constructs are too weak to be handled unless utilizing strong cross-linking agents whereas they are usually not favourable for cell printing process. Examples of bioprinting approaches utilizing solid scaffolds as support would take reference from recent studies presented by Kang *et al*.^17^ and Jung *et al*.^18^. 3D printing of solid polycaprolactone (PCL) scaffolds was accomplished simultaneously with the cell-laden hydrogels. Stacking of the bioprinted constructs becomes feasible when they incorporated 3D printing of scaffolds into bioprinting. However, PCL degrades over a long period of ≥2 years^19^ and the cell number per unit volume of the bioprinted construct is quite low^17^,^18^. In addition, thermoplastic polymer was melted at elevated temperatures (e.g. PCL at ≥60 °C) and then deposited onto the previous layer containing cell-laden hydrogel, which may be harmful to some cells^20^.

Tissue spheroids or tissue strands have also been used in 3D bioprinting^14^,^15^. These units of spheroids or strands can be fused in bioprinting, allowing the maximum possible initial cell density, without using any scaffolds^15^. An example of scaffold-free bioprinted construct is the vascular tubular tissue created by Itoh *et al*.^21^. One distinct advantage of the bioprinting strategy using scaffold-free spheroids is the ability to expedite tissue organization^14^. However, the scaffold-free bioprinting process requires rapid tissue maturation so that the shrinkage of the construct is minimized, and the shape, tissue composition and integrity are well-controlled^15^. Furthermore, a very high initial cell number is needed for the fabrication of scaffold-free tissue spheroids/strands in the large tissues’ bioprinting. For example, the aforementioned printed small vascular tubular tissue of 1.5 mm in diameter and 7 mm in length needed an initial cell number of ~1.25 × 10^7 ^21. Scaling up tissue constructs will consume a tremendous number of cells that must be extracted from the patient; otherwise the primary cells have to be greatly expanded in laboratory, which is unrealistic currently as each type of cell has its passage limitations.

Considering what is feasible for 3D bioprinting nowadays, in this work, we are presenting a micropipette extrusion-based bioprinting method using cell-laden microscaffold-based bioinks. Polymer microcarriers such as microspheres are commonly utilized as injectable biomaterials for clinically relevant applications^22^,^23^. Solid polymer microcarriers was also used in biofabrication for bone tissue engineering^24^. Most of the normal mammalian cells need substrates to adhere and proliferate^25^. Here the highly porous microscaffolds provide high specific surface areas so that they allow the anchorage-dependent cells to attach, infiltrate and grow before extrusion-based printing. By exploiting this property, the cells seeded on the microspheres will be expanded in stirred or perfused culture, and form cell-laden microspheres (CLMs) without further passaging. These CLMs together with thin hydrogel encapsulation act as the bioink for 3D bioprinting. The hydrogel lubricates the CLMs during printing and glues the CLMs together after printing upon gelation (Fig. 1). The addition of type I collagen in the hydrogel further improves its cells adhesion during culture. Micropipette^26^ was used in the printing in order to achieve tightly packed constructs.

## Results

### Bioink fabrication

#### Microspheres fabrication

**Figure 2:**
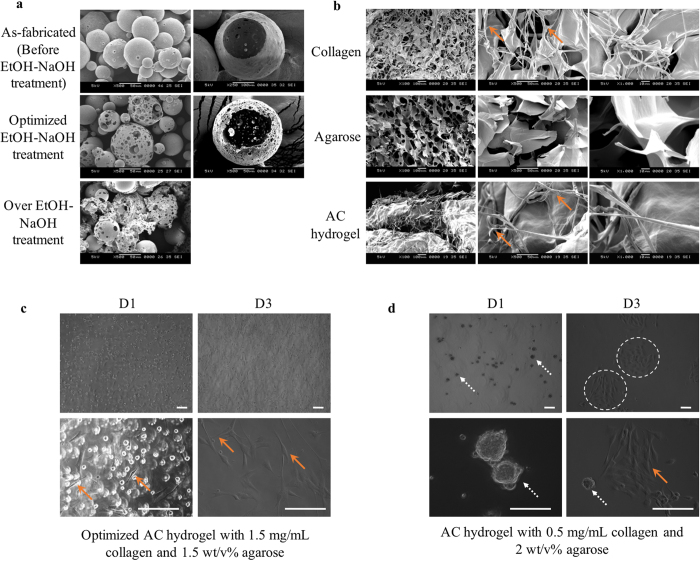
Bioink optimization. (**a**) SEM images of PLGA microspheres as-fabricated, optimized treatment (5 min), and over treatment (10 min) of EtOH–NaOH solution. Images on the right show the fractured cross-section of the corresponding microspheres. Most of the over-treated microspheres were fragmented. (**b**) SEM images showing microarchitecture of 1.5 mg/ml collagen, 1.5 wt/v% agarose and AC hydrogels. Collagen fibrils (arrows) can be observed in collagen and AC hydrogel, but not in agarose. OM images of myoblasts C2C12 cast in AC hydrogels with (**c**) 1.5 mg/ml collagen and 1.5 wt/v% agarose (optimized and used in this work) and (**d**) 0.5 mg/mL collagen and 2 wt/v% agarose, after 1 day and 3 days of culture. Images shown were taken from the top surface of the hydrogel to avoid imaging of cells attached to the tissue culture polystyrene (TCPS). AC hydrogel with low concentrations of collagen lacks RGD for cell attachment, e.g. cells colonization (dotted arrows) occurred in the hydrogel with 0.5 mg/ml collagen and 2 wt/v% agarose. Although most of the cells became elongated (arrows) after 3 days of culture, they remained in colonies (dashed circles). Meanwhile AC hydrogel with lower concentrations of agarose (≤1.5 wt/v%) are not favoured in the printing process as gelation of hydrogel in each layer are slow. Bioprinting can be achieved smoothly with fast gelation using optimized AC hydrogel. Cells attained normal morphology (arrows) in the optimized AC concentration. Scale bars, 200 µm.

Poly(D,L-lactic-co-glycolic acid) (PLGA) was chosen as a suitable material for fabricating the biodegradable microspheres because its safety in clinically relevant applications has been well established^27^. The PLGA microspheres fabrication was optimized as shown in Fig. 2a. An ethanolic sodium hydroxide (EtOH-NaOH) treatment time of 5 min was identified as the optimum for enlarging the pore size of the microspheres without compromising their structural integrity. The microspheres fabricated ranges from 60 to 130 μm. They possessed an extremely high porosity with an average pore size of 7.3 μm (ranging from 1.0 to 20.9 μm). Calculation were performed excluding multiple submicron pores concurrently observed on the microspheres.

#### Thermoresponsive agarose-collagen composite hydrogel (AC hydrogel) fabrication

Lubricating AC hydrogel^28^ was used as a thin encapsulating material enveloping the microspheres for ease of printing. At below ~20 °C, the AC hydrogel will work as an adhesive that assembles the microspheres into a pre-defined shape. Ice was placed under the build platform to expedite adhesion. The AC hydrogel was selected among other hydrogels because it has good extrudability; enables an immediate gelation upon cooling; and allows formation of collagen fiber networks within the printed construct during culture at 37 °C. Collagen fibrils were formed in the AC hydrogel (Fig. 2b) after incubation at 37 °C for 24 hrs. We have tested a variety of AC hydrogels with different combinations in order to achieve optimal printing resolution, dispensing uniformity, solidifying time, and stacking ability while not sacrificing its cell viability and spreading (Fig. 2c,d).

### Cell attachment and viability

**Figure 3:**
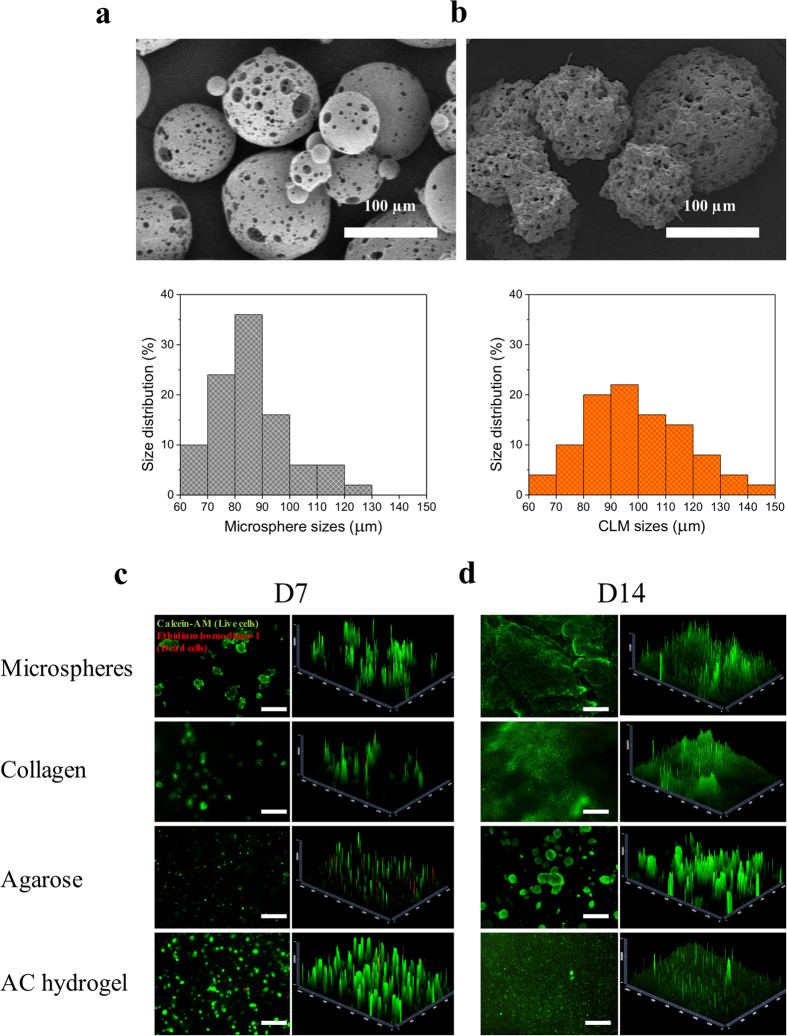
Characterization of the bioink. SEM images showing (**a**) the PLGA microspheres and (**b**) the CLMs. The size distributions of the microspheres and the CLMs were given under the respective SEM image. (**c**) Day 7 and (**d**) day 14 L929 cell viabilities on PLGA microspheres, 1.5 mg/mL collagen, 1.5 wt/v% agarose and AC hydrogel. First set of fluorescence images (left) are the live (green)/dead (red) images of the cellular construct captured by the fluorescence microscope; the graphs on the right show the 2.5D reconstruction of the fluorescence images for a clearer visualization of the positions of live and dead cells. Scale bars, 500 μm.

To determine cell attachment and proliferation in PLGA microspheres, we seeded mouse fibroblasts L929 cells into the microspheres using stirred culture. Cells attached well on the microspheres as shown in Fig. 3a,b. The highly porous PLGA microspheres have good cell affinity as expected and thus high cell density CLMs were produced. The CLMs sizes ranged from 60 to 150 μm as shown in Fig. 3b.

The cell viabilities of L929 on the microspheres and hydrogels were accessed through live/dead assay on day 7 and day 14 of culture (Fig. 3c,d). Both the fabricated PLGA porous microspheres and the AC hydrogel are highly biocompatible, showing more than 90% viable cells after 1 and 2 week(s) of culture. Cells were spread out in the AC hydrogel after 14 days of culture, similar to that of collagen. By contrast, in agarose hydrogels, cells were rounded and formed into big cell aggregates.

### Micropipette-based bioprinting

**Figure 4:**
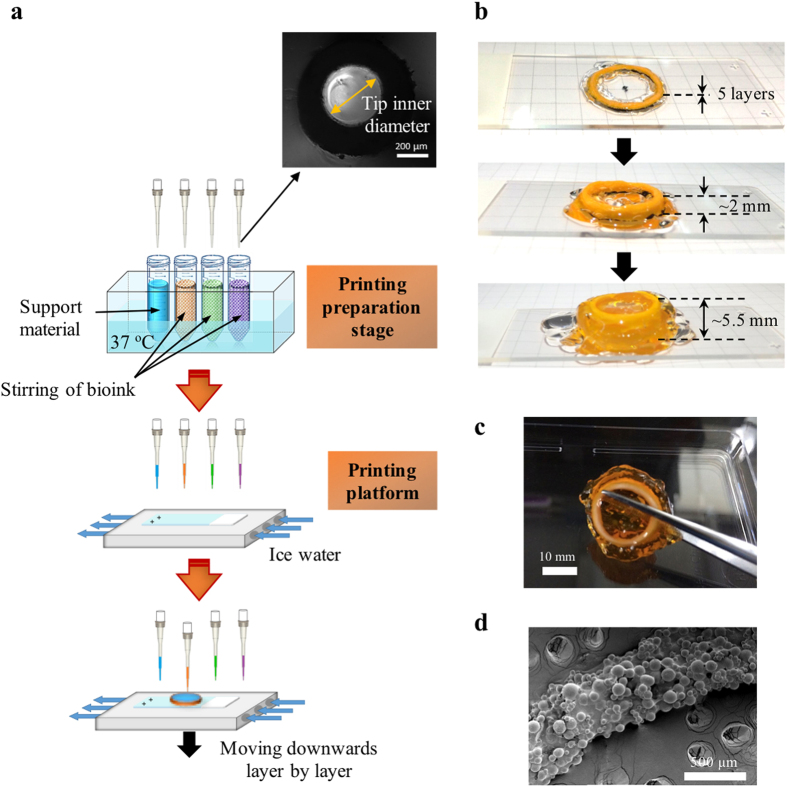
Schematic of an automated bioprinting system and the 3D-printed prototype. (**a**) The system should consist of four major units: (i) a 3-axis robotic controlled dispensing system using micropipette tips, (ii) a printing preparation stage with boxes of new micropipettes and bioinks under stirring and temperature control (37 °C), (iii) an ice-water-chilled printing platform, and (iv) a closed chamber with humidifier, UV lamp, and trash container. Before printing, the chamber can be sterilized with UV lamp. Bioinks can be prepared prior to loading into the printing preparation stage. Printing is conducted on the chilled platform where the hydrogel will glue the microspheres into designed shapes layer by layer. Tips will be removed into the trash container after layers of printing and new tips will be fetched onto the dispensing system before drawing the bioinks for subsequent printing. Inset shows the OM image of the micropipette tip. (**b,c**) Pictures of 3D-printed tubular construct with gelatin (transparent) as support. Constructs were printed on superfrost plus microscope slides with a dimension of 25 mm × 75 mm. (**d**) SEM image of printed construct showing tightly packed microspheres.

Extrusion-based technology permits the precise fabrication of complex structures and facilitates the patterning of multiple types of cells^3^,^12^. Instead of the commonly used extrusion approaches, we propose a micropipette-based extrusion method, as illustrated in Fig. 4a, for 3D printing of tightly packed CLMs. The bioink, i.e. CLMs encapsulated in thin AC hydrogel, was made by adding the hydrogel into the CLMs, followed by pipetting, centrifuging and removal of supernatant. The packing of the microspheres per unit volume of the bioink was ~74%, by assuming densest packing of monodispersed microspheres^29^. Bioink with different cell types can be prepared in separate centrifuge tubes and then loaded into a printer, with gentle stirring and precise temperature control. Utilizing this design will prevent CLMs from fusing before the printing process. Each micropipette tip is capable of drawing up some bioink from respective centrifuge tube concurrently for printing of a few layers. It is noted that clogging of tips during printing and the resultant waste of bioink are the main limitations for extrusion-based bioprinting which also includes our method. However, clogged tips can be replaced in an automated way when using micropipette-based extrusion printing. In fact, there have been some commercial machines that feature automated pipetting systems, which can be implemented into a bioprinter. Although it may induce wastage of bioinks when removing the clogged tips, this bioprinter is able to print large 3D tissues consecutively in a humidified closed chamber with the reduced risks of contamination.

The extrusion-based printing was achieved manually in this work, but it is obvious that complex 3D biological constructs can be bioprinted using an automated printer. 3D printing of a tube with a mean diameter of 15.0 mm and height of ~5.5 mm was carried out using the micropipette extrusion method as shown in Fig. 4b,c. Here orange-coloured food dye was added to AC hydrogel for illustration purpose. Extrusion of the bioink can be performed effectively, with a fast gelation upon printing to the cold build platform. High concentration of gelatin hydrogel (transparent) was used as a support material. It gels at temperatures lower than 37 °C, forming a firm support to the construct. Gelatin was removed during subsequent culture at 37 °C as it liquefies. As shown in Fig. 4d, tightly packed PLGA microspheres can be printed using the micropipette extrusion method, with a printing resolution of ~500 μm.

### Compressive properties

**Figure 5:**
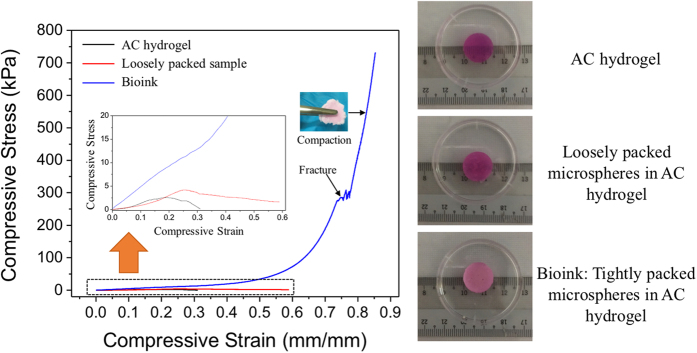
Compressive stress-compressive strain properties. Compressive stress-compressive strain curves for the AC hydrogel, the loosely packed samples, and the bioink. Pictures on the right show the representative samples used for compression tests.

In order to show the advantage of the bioprinted construct in terms of its mechanical properties, compression tests of the cast AC hydrogel and bioink (acellular PLGA microspheres tightly packed in AC hydrogel; ~74%^29^) were conducted. Cast samples with PLGA microspheres that were loosely packed in AC hydrogel (loosely packed samples; ~20 v/v%) were prepared as comparison. The cylindrical samples were cast on ice, followed by incubation in cell culture media at 37 °C oven for 3 days before testing. Figure 5 shows the corresponding compressive stress–compressive strain curves of the samples. The AC hydrogels were fractured under low deformation as expected. It failed at an average stress of 2.7 kPa and strain of ~30%. The loosely packed samples were fractured at a higher average stress of 4.4 kPa and strain of ~60%. Meanwhile, the cast bioink broke at an outstandingly higher stress of 270.6 kPa and at a high strain of ~75% compared to the other types of samples. These samples continued to compact after fracture as shown in the inset. The average compressive moduli of the AC hydrogel, the loosely packed samples and the bioink were computed to be 22.5 kPa, 29.1 kPa and 47.6 kPa, respectively.

### Cell patterning, viability and proliferation

**Figure 6:**
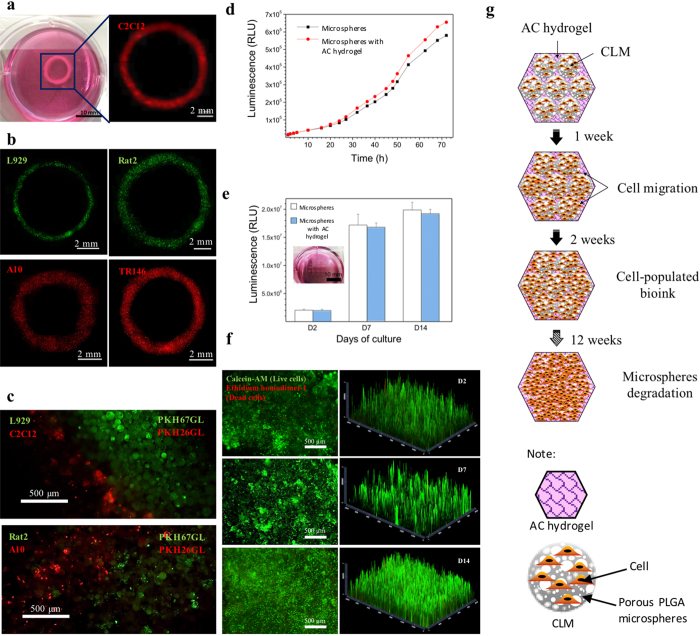
Observation of as-bioprinted ring constructs using different types of cells and cell viability of bioprinted constructs. (**a**) Photograph and fluorescence image of a printed ring using C2C12 cells labelled with red fluorescent cell linker PKH26GL. (**b**) Fluorescence images of printed ring constructs with L929, Rat2, A10 and TR146 cells. L929 and Rat2 cells were labelled by green fluorescent cell linker PKH67GL, and A10 and TR146 cells were labelled with red fluorescent cell linker PKH26GL. The ring-shaped fluorescence images were combined from multiple images of each samples captured under fluorescence microscope. Images (**c**) reveal patterning of L929 cells (green) together with C2C12 cells (red) and Rat2 cell (green) side-by-side with A10 cell (red) achieved through the printing (**d**) Real time cell viability and proliferation of printed 3D constructs show that the number of cells was continuously increased over 72 hrs. There were no significant differences between the control and the printed constructs (n = 3). (**e**) 3D cell viability within the printed construct on the day 2, 7, and 14 of culture after printing shows the cell growth over 14 days. Insert shows the printed construct used for this study. (**f**) L929 cell viability was over 90% on day 2, 7 and 14 after printing. First set of fluorescence images (left) are the live (green)/dead (red) images of the construct captured by a fluorescence microscope; another set of graphs (right) show the 2.5D reconstruction of the fluorescence images by using ZEN microscope software for a clearer visualization of the locations of the live and dead cells. (**g**) Schematic illustration of prediction of the cell growth patterns in bioprinted constructs. The PLGA did not show autofluorescence. All fluorescent signals were originated from the labelled viable cells or stained cells.

We verified the current 3D bioprinting method via patterning different types of cells. By labelling fibroblasts L929 and Rat2 with green fluorescent cell linker, and the myoblasts C2C12 and A10 and the epithelial TR146 with red fluorescent cell linker, we printed the different cells into 3D rings (Fig. 6a,b). We also demonstrated the delivery of two populations concurrently in each layer of printing as illustrated in Fig. 6c.

To determine cell viability after extrusion, we assessed the survival of L929 from 1 to 72 hr(s). The printed cells were found to survive well after printing and continued to proliferate over the 72 hrs period, similar to the proliferation of control wells (Fig. 6d). For a longer term study of printed cellular construct, the cell viability was accessed after culturing the bioprinted constructs for 2, 7 and 14 days. (Fig. 6e). The printed cells continued to proliferate for 14 days after printing, with no significant difference when compared to the control wells.

**Figure 7:**
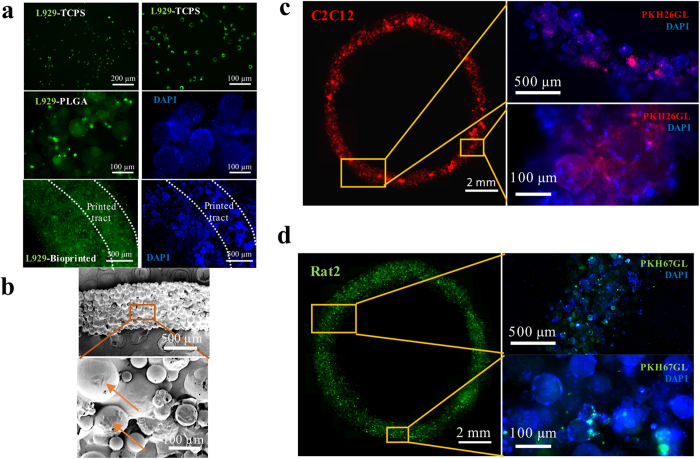
Characterisation of bioprinted ring constructs. (**a**) Fluorescence images of L929 cells labelled by green fluorescent cell linker PKH67GL cultured on TCPS, PLGA microspheres, and printed construct. High cell density could be observed on the printed construct. (**b**) SEM images of the printed construct using L929 cells. The construct was fully covered with cells. Obvious cells are indicated by arrows. Fluorescence images of printed constructs using (**c**) C2C12 cells labelled with red fluorescent cell linker PKH26GL and (**d**) Rat2 cells labelled by green fluorescent cell linker PKH67GL after 3 days of culture. The fluorescent signals proved that the cells are still viable. Blue fluorescence images (right) in (**a**,**c**,**d**) show DAPI (nuclei) staining of the corresponding samples.

Imaging by live/dead cell assays showed ≥90% cell viability for 2, 7 and 14 days after extrusion (Fig. 6f). 2.5D reconstruction using Zen software presents clearer position of live and dead cells. Figure 6g illustrates the cell growth patterns within the bioink, which can be drawn based on the live/dead assay results. Initially, cells were only on the microspheres, forming thin spikes of green signals in the live/dead 2.5D reconstruction graph. After 7 days of culture, the cells continued proliferating within the CLM; and started migrating out to the AC hydrogel. Homogeneous green signals from the live/dead graph after 14 days of culture indicate that cells were populating the whole bioprinted construct. Microspheres are supposed to degrade after 12 weeks of culture based on the hydrolytic degradation results (Supplementary Fig. S1a,b). From Fig. 7a,b, viable cells can be observed on the bioprinted tracts immediately after printing. After 3 days of culture, the constructs were still filled with live cells as shown in Fig. 7c,d. These results indicate that the printed construct maintained cell viability during the bioprinting process and provided a favourable microenvironment for cell proliferation.

## Discussion

PLGA microspheres were used in this work, but other injectable microscaffolds/microcarriers e.g. microspheres and microfibrils^30^, fabricated from biodegradable polymeric materials such as polyesters^23^, polypeptides^31^, polysaccharides^32^, and their combinations^33^, could serve the same purpose in this context. The scaffolds provide 2D surfaces with pseudo-3D environment for the anchorage-dependent cells to adhere and proliferate. At this instant, scaffolds fabricated from biomaterials with a controllable degradation time frame are preferred. PLGA microspheres could degrade within 12 weeks hydrolytically *in vitro* at 37 °C (Supplementary Fig. S1a,b). Also, a highly porous microscaffold is favourable compared to a full solid scaffold in order to pack high density of cells into the scaffold and achieve expedite degradation after printing. As amorphous PLGA has a glass transition temperature (T_g_) higher than 37 °C (Supplementary Fig. S1c), the microspheres could remain non-cohesive^23^ for smooth printing without clogging.

By using cell-laden microscaffolds in bioprinting of a 3D construct, a lower initial cell density is achievable as compared to that required by conventional cell-laden hydrogels or tissue spheroids/strands. For example, when using highly porous microspheres, initial density of ~2.7 × 10^4^ cells/mm^3^ is sufficient. By contrast, cell-laden hydrogel printing requires a cell density of ~1.7 × 10^5^ cells/mm^3^ while tissue spheroids printing entails ~1.8 × 10^6^ cells/mm^3^ (calculated in Supplementary Data file S1). With the readily available biomaterials and the mature technology in fabrication of the microscaffolds, microscaffold-based bioprinting becomes easy to implement. Size distribution of the microscaffolds can be controlled well by varying the fabrication parameters. Furthermore, the polymeric microscaffolds were found to be stable over a relatively long period after fabrication if properly stored. With the support of these biodegradable microscaffolds, printed constructs can undergo a slower and more controllable process of tissue maturation as compared to the scaffold-free constructs.

The microspheres size range was selected with good reasoning. The chosen as-fabricated microspheres size range (before EtOH-NaOH treatment: sieved to ~90–150 μm) is ideal for this study as pores’ enlargement using EtOH-NaOH is limited by the microsphere size, especially when microsphere sizes are small. It is known that most of the mammalian cells have a diameter of ~10 μm when rounded up after detachment. Cell infiltration would be impossible if the pores on the microspheres are too small, which would reduce the cell density per microsphere. The optimized pore size range was found to be ~0.1–20 μm. Further pore enlargement treatment causes microsphere fragments as shown in Fig. 2a. Submicron pores assist in nutrient exchange while the macropores allow cells to infiltrate. After enlargement of pores, the microspheres size became smaller (60–130  μm) due to the “etching effect” of the EtOH-NaOH. Obviously, a higher printing resolution could be realized by using smaller microspheres. Hence the adopted microspheres size range was determined by a trade-off between printing resolution and cell density. Of note is that size of CLMs became bigger (60–150 μm) because of the slight swelling of PLGA microspheres after submerging in cell culture media at 37 °C^34^. Cells seeded on the microspheres also contribute to the larger size of the CLMs when compared to the acellular microspheres. The printing resolution achievable here is ~300–600 μm depending on the printing speed, which is of no difference to the current bio-printable resolution^35^,^36^,^37^.

Agarose hydrogel is not uncommon in TE scaffolds fabrication^38^,^39^. It has also been used in bioprinting but usually printed as molds^14^, thanks to its superb thermal properties, extrudability, and non-stickiness. Agarose exhibits pronounced hysteresis between the gelling and the melting^40^, gels at below 18–42 °C and re-melts at above 60–90 °C. The gelling and melting temperatures depend on its end groups. Low gelling point agarose was chosen in this work so that the hydrogel remains as liquid at a printing temperature of 37 °C. Despite its advantages, agarose is a polysaccharide that lacks the arginyl-glycyl-aspartic acid (RGD) for cell attachment and proliferation. Type I collagen is added as a favored adhesive substrate to make the AC composite hydrogel more cell-affinity. Cell spreading is promoted using the AC hydrogel as compared to the agarose. The AC hydrogel with optimized concentrations of agarose and collagen shows good printability and cell attachment compared to other concentrations of AC hydrogels. Here, the printability means good extrudability; enables immediate gelation upon extrusion onto cold platform; and allows formation of collagen fiber networks within the printed construct during subsequent culture at 37 °C. High concentration gelatin was chosen as a support material due to its attribute as a biocompatible hydrogel gelling at low temperatures. It is commonly used as support material in bioprinting^41^ as it melts at culture temperature of 37 °C, allowing easy removal of the temporary support material once the bioprinted construct can self-support.

The current bioprinting strategies include extrusion-based, microvalve-based, laser-based, and inkjet-based printing^2^,^42^. Microneedle, tapered tip, or nozzle based syringe extrusion methods were widely applied in the reported extrusion-based bioprinting approaches^7^,^12^,^43^,^44^. We herein found that the micropipette-based extrusion method is a good approach to print tightly packed PLGA microspheres. The tightly packed microspheres encapsulated in thin AC hydrogel samples could sustain a much higher stress and strain than both the AC hydrogel and the loosely packed samples. The mechanical strength of the bioink was dramatically improved by more than 100 times when compared to that of the AC hydrogel. Meanwhile, when the microspheres are loosely packed in AC hydrogel, the mechanical strength of the construct was increased only by 1.5 times compared to the AC hydrogel. The compressive moduli of the tightly packed microspheres samples were superior compared to the AC hydrogel. The improvement in mechanical properties was attributed to the packing of strong polymer microspheres.

This 3D bioprinting approach, when compared to pure hydrogel bioprinting, allows a better stacking ability for the fabrication of 3D constructs. A volumetric 3D tissue can be built up in a few layers. With cushioning or shielding effect from the microspheres, there will be less shear stress-induced cell damage during extrusion-based printing process. The printed construct has been proven to provide a suitable 3D environment for different types of cells to grow. For example, bioprinted rat smooth muscle A10 cells can be clearly seen under SEM (Supplementary Fig. S2a) after 3 days of culture. The F-actin architecture was observed from the printed A10 construct but it was random with no preferential alignment, as opposed to the control (Supplementary Fig. S2b,c). This can be attributed to the random distribution of the isotropic microspheres. It is suggested that cell-laden microfibrils or microfibers^45^ can be used in bioprinting to provide anisotropic alignment when an aligned tissue is needed such as with neural^46^ and gastrointestinal tract tissues^47^,^48^.

Multipotent mesenchymal and pluripotent stem cells can be expanded by culturing with the microscaffolds^49^,^50^. By using microscaffolds in 3D bioprinting, well-established surface treatment on scaffolds can be applied to enhance cell affinity^51^. Bioactive factors can be added into the scaffolds and thus effectively control the stem cell fate. Levato *et al*. used a collagen-functionalized solid microcarrier-based approach to print bilayered osteochondral models for the bone compartment^24^. They proved that the microcarriers facilitated cell adhesion and supported bone cells differentiation by mesenchymal stem cells. The incorporation of proteins and drugs such as growth factors, antibody, and cell adhesion peptides into the microscaffolds can be realized for a sustainable release system in the printed construct. Poldervaart *et al*. printed bone morphogenetic protein 2 (BMP-2) and vascular endothelial growth factor (VEGF) loaded gelatin microparticles for bone regeneration^52^,^53^. A graded concentration of these molecules can also be accomplished in printing. In addition, the microscaffolds can be functionalized, for example by encapsulation of magnets for *in vivo* imaging of the implanted organ^54^.

In this study, we have shown the ability of bioprinting CLMs by using the micropipette extrusion-based method. Tight packing of microspheres was achieved. However, both resolution and speed of extrusion-based printing method are still not in the desired range for large tissue printing. Indirect printing of the construct, e.g. printing of sacrificial moulds before extrusion of the bioink, can improve the printing resolution due to dimensional constriction whilst within the limitation of the microsphere sizes. We also suggest that 3D bioprinting of microscaffolds is not restricted to extrusion-mode. For instance, CLMs could be tightly packed in photocurable hydrogels, followed by photo-curing layer by layer for highly efficient printing of tissue constructs^55^. To further explore the possibility of the microscaffolds bioprinting approach, future strategies could be extended to utilize new material combinations, produce different forms of microscaffolds, and develop novel printing methods to assemble CLMs.

## Conclusions

In conclusion, we presented a 3D bioprinting technique using micropipette extrusion-based method for the biofabrication of 3D living multicellular tissues. The bioink is constituted of PLGA porous microspheres and thin encapsulation of AC hydrogel, whereby the microspheres were cell-seeded and cell-expanded in a stirring flask before printing. The use of cell-laden microscaffolds instead of the conventional scaffold-free cell-laden hydrogels and tissue spheroids/strands as the building block in bioprinting can potentially solve the problem of cell source shortage. Here different types of cells were successfully printed. Cells were viable after printing, and they continue to proliferate with culture time. The bioprinted construct possessed high biocompatibility, with cell viability of more than 90% after culturing for 2, 7, and 14 days. Furthermore, the mechanical strength of the construct was greatly improved by more than 100 times when compared to the AC hydrogel. By coupling the advantages of solid scaffolds with the new 3D bioprinting concept, this work enables the fabrication of multiscale and multicellular 3D tissue constructs, and thus providing a promising alternative in bioprinting of clinically relevant tissue replacements and functional *in vitro* biological models.

## Methods

### Microsphere fabrication

Porous PLGA with DL-lactide and glycolide in a 50/50 molar ratio (PURASORB® PDLG 5010) microspheres were fabricated via a modified double emulsion method^22^,^23^. PLGA solution (20 wt/v% in dichloromethane (DCM)) and salt solution (1 mL of 10 × PBS (Sigma) mixed with 4 mL of 0.3 wt/v% poly(vinyl alcohol) (PVA; Sigma) solution in double-distilled water (ddH_2_O)) were prepared. The salt solution was poured onto 5mL of PLGA solution, homogenized at 10,000 RPM for 2 min, and then quickly poured into a magnetically stirred 0.3 wt/v% PVA solution at 1000 RPM. The double emulsion was stirred overnight. Microspheres were rinsed thrice with ddH_2_O and vacuum filtered. The microspheres were sieved by using sieves with nominal aperture of 88 and 149 μm. Pores of the microspheres were enlarged by treating the microspheres with ethanolic sodium hydroxide (0.25 M NaOH (FLUKA):70 v/v% ethanol (EtOH) ratio of 3:7). The microspheres were vacuum filtered, rinsed thrice with ddH^2^O, vacuum filtered, freeze-dried for ~24 hrs, and then kept at 4 °C.

### Hydrogel Preparation

The AC hydrogels are made of agarose and collagen mixed into high glucose DMEM medium (Gibco). Stock agarose solutions were prepared by dissolving 3 wt/v% agarose Type IX-A with ultra-low gelling temperature (Sigma) in DMEM by autoclaving the mixture for 15 minutes at 100 °C the solution was then brought to 37 °C. Stock collagen solutions were prepared on ice immediately prior to use by neutralizing collagen type I solution (3.34 mg/ml rat tail collagen, Corning®) with dropping 1 M NaOH to bring the pH to 7.4. AC hydrogels were prepared by mixing agarose and collagen stock solutions with additional warm DMEM in appropriate volumes to create composite hydrogel.

Gelatin hydrogel was prepared by dissolving 10 wt/v% gelatin Type A with gel strength 300 (Sigma) in DMEM by autoclaving the mixture for 15 minutes at 100 °C. The solution was brought to 37 °C in a water bath.

### Characterization

The surface morphology of microspheres was viewed under a scanning electron microscope (SEM, JEOL JSM-5600LV). Cross-section was captured after fracturing the microspheres. The sizes of the microspheres, microsphere pores and CLMs were measured using Image J software. Hydrogels were prepared as previously described, casted on ice, followed by incubation at 37 °C for 24 hrs, and then freeze-dried prior to SEM imaging.

### Mechanical characterization

The uniaxial compressive stress–strain measurements were performed on cylindrical samples after submerging them in DMEM supplemented with 10 v/v% fetal bovine serum (FBS; Gibco) and 1 v/v% antibiotic/antimycotic solution (Gibco) at 37 °C for 3 days. Instron 5566 universal testing machine with a load cell of 100 N was used for the testing at a crosshead speed of 1 mm min^–1^. The cylindrical AC hydrogel, loosely packed PLGA microspheres (~20% v/v) in AC hydrogel and tightly packed PLGA microspheres in AC hydrogel samples (n = 3) were 13–15 mm in diameter and ~4 mm in thickness.

### Cell culture

All cells were cultured in their respective culture media and maintained in a humidified tissue-culture incubator at 37 °C and with 5% CO_2_. L929 and Rat2 were labelled with green fluorescent cell linker (PKH67GL; Sigma); and C2C12, A10 and TR146 were labelled with red fluorescent cell linker (PKH26GL; Sigma) according to the manufacturer’s instructions prior to cell seeding on microspheres.

### Cell seeding on microcarriers

0.3 g of microspheres were sterilized by immersing in 70 v/v% EtOH at 4 °C for 5 hrs. The microspheres were washed thrice with PBS before suspended in cell culture media in a 125 mL siliconized Techne biological stirrer flask (Bibby Scientific Limited). A total cell number of 2.5 × 10^7^ were suspended into the flask. Pre-warmed medium was added into the stirrer flask to make a total solution of 40 mL, which the culture is stirred intermittently for 2 min every 30 min at the speed of 30 RPM for 6 hrs. Total media volume of the culture was increased to 125 mL and continuously stirred at 60 RPM for another 20 hrs.

### Bioink preparation

Preparation of bioinks and the subsequent printing process were conducted in a clean room to ensure a sterile environment for all the transfers. The AC hydrogel and the support material gelatin hydrogel were kept in a water bath at 37 °C. The microspheres were centrifuged and the supernatant was removed. The AC hydrogel was quickly added to the microspheres and gently pipetted prior to centrifugation and supernatant removal. The bioink, i.e. microspheres coated with AC, was kept in water bath at 37 °C prior to printing.

### Constructs printing

Printing of constructs was accomplished using a simple, hand-held printing process using micropipettes (size of 1–10 μL). An ice platform was prepared to glue the microspheres together. Prior to printing, all the equipment was sterilized by spraying with 70% EtOH followed by 1 hr of UV bath. Superfrost plus microscope slides (Thermo Scientific) were put onto the ice platform and the bioink was withdrawn into the micropipette. The bioink was then extruded into the desired shape on the glass slides and the 2.5D construct was formed immediately. A 3D construct was built after a layer-by-layer printing. In order to build a tall, complex construct, gelatin hydrogel was utilized as a support material, whereby it is laid down layer by layer to support the biological structure.

### Cell viability, proliferation, attachment and immunofluorescence studies

Cell viability of L929 on the microspheres, hydrogel, and printed constructs were accessed using live/dead assay (Molecular Probes) after 2, 7 and 14 days of culture. Fluorescence microscopy (Zeiss Axio Vert. A1) was used to evaluate the live/dead staining of cells in the samples. 3 days’ cell proliferation of the 3D printed constructs was determined by the RealTime-Glo™ MT Cell Viability Assay (Promega). The cells seeded microspheres were used as a control. The number of viable cells was determined in culture by measuring the intensity of luminescence signals of assay solutions at several time points from 1 hr up to 72 hrs after culture using a microplate reader (Ultra Evolution, Tecan). Days 2, 7, and 14 cell viability within the printed 3D constructs were studied using CellTiter-Glo® 3D (Promega). 100 μl of the assay solution from each sample was placed into the wells of a white 96-well plate and luminescence intensity was measured using microplate reader. All the assays were conducted according to the manufacturer's instruction. Cellular samples to be observed by SEM were rinsed with PBS and fixed with 2.5% glutaraldehyde overnight at 4 °C. Following PBS rinses, the samples were dehydrated through a series of graded EtOH solutions, air-dried, gold-coated and observed under SEM. NucBlue® Live ReadyProbes® Reagent (Molecular Probes) was utilized to image the nuclei of the constructs by dropping 2 drops of reagent per mL of the culture media. The samples were incubated at room temperature for 20 min before imaging with a fluorescent microscope.

The more detailed experimental procedures and data analysis can be found in the Supplementary Information.

## Additional Information

**How to cite this article**: Tan, Y. J. *et al*. Hybrid microscaffold-based 3D bioprinting of multi-cellular constructs with high compressive strength: A new biofabrication strategy. *Sci. Rep.* **6**, 39140; doi: 10.1038/srep39140 (2016).

**Publisher's note:** Springer Nature remains neutral with regard to jurisdictional claims in published maps and institutional affiliations.

## Supplementary Material

Supplementary Information

## Figures and Tables

**Figure 1:**
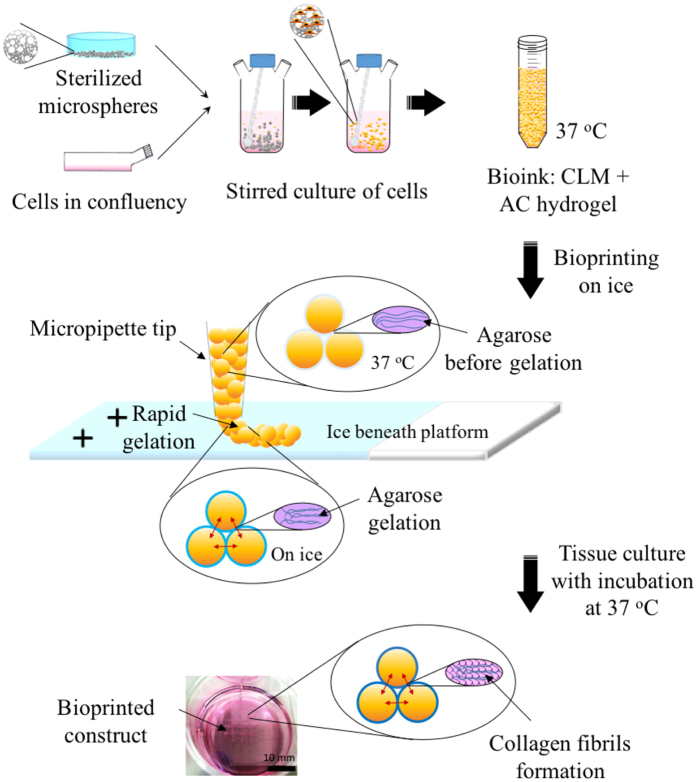
Schematic illustration of the bioprinting process. Cells (indicated in orange) are seeded onto the porous PLGA microspheres. After stirred culturing, cells infiltrate and proliferate into the microspheres, producing CLMs. These CLMs are encapsulated with a thin layer of thermoresponsive AC hydrogel for bioprinting on a chilled platform, where agarose gelation occurs. Collagen fibrils are formed after culturing the construct at 37 °C.
