# Supplementary material for: Hybrid microscaffold-based 3D bioprinting of multi-cellular constructs with high compressive strength: A new biofabrication strategy
Source: Sci Rep. 2016 Dec 14;6:39140. doi: 10.1038/srep39140 (PMC5155425; doi:10.1038/srep39140)
Supplement: Supplementary Information [file srep39140-s1.pdf]

Supplementary Information.

Hybrid microscaffold-based 3D bioprinting of multi-cellular constructs with high compressive strength: A new biofabrication strategy

Yu Jun Tan, Xipeng Tan*, Wai Yee Yeong*, Shu Beng Tor*

Methods

Microsphere fabrication: Porous poly(DL-lactide-co-glycolide) (PLGA) microspheres were fabricated via a modified double emulsion method. In this work, PLGA (PURASORB® PDLG 5010, Corbion, Netherland) with DL-lactide and glycolide in a 50/50 molar ratio was used. 5 mL of PLGA solution with 20 wt/v% of PLGA dissolved in dichloromethane (DCM, AppliChem GmbH, Germany) was prepared. Salt solution was prepared separately by mixing 1 mL of 10× phosphate buffered saline (PBS; P5368; Sigma–Aldrich, St. Louis, MO, USA) into 4 mL of 0.3 wt/v% poly(vinyl alcohol) (PVA; Mowiol® 8-88; Sigma–Aldrich, St. Louis, MO, USA) solution. Both the solutions were previously prepared in a double-distilled water (ddH_2O). The salt solution was carefully poured on the PLGA solution whereby the two-layered solution were left to sit for 2 min prior to emulsification. Emulsification was initiated by homogenizing the solution at 10, 000 rpm for 2 min using Wiggenhauser Homogenizer D-500. The primary water in oil (w/o) emulsion was then quickly poured into a magnetically stirred 200 mL of 0.3 wt/v% PVA solution at 1000 rpm. The resultant water-in-oil-in-water (w/o/w) double emulsion was left stirring overnight to allow microspheres hardening and DCM evaporation. The microspheres were harvested followed by three ddH_2O wash and vacuum filtration. The microspheres were sieved by using sieves with nominal aperture of 88 and 149 μm. Subsequently, the pores of the microspheres were further enlarged by treating the microspheres with ethanolic sodium hydroxide with 0.25 M NaOH (35274 FLUKA; Sigma–Aldrich, St. Louis, MO, USA): 70 v/v% absolute ethanol (EtOH; Merck, Germany) in a ratio of 3:7. Both the solutions were diluted with ddH_2O to the desired concentrations prior to mixing.

Briefly, one portion of microspheres was suspended in ten portions of EtOH–NaOH solution with magnetically stirring at 1000 rpm for 5 min. The microspheres were vacuum filtered, followed by three ddH_2O rinse. After final vacuum filtration, the microspheres were dried in a freeze-drier (ScanVac CoolSafe Freeze Drying, LaboGene™, Denmark) for ~24 hrs and then kept in a fridge at 4 ^{o}C for further usage.

Hydrogel Preparation: The hydrogel to glue the microspheres is made of agarose and collagen mixed into high glucose DMEM medium (Gibco, UK). Stock agarose solutions were prepared by dissolving 3 wt/v% agarose Type IX-A with ultra-low gelling temperature (A2576; Sigma–Aldrich, St. Louis, MO, USA) in DMEM by autoclaving the mixture for 15 minutes at 100 ^{o}C; the solution was then brought to 37 °C in a water bath. Stock collagen solutions were prepared on ice immediately prior to use by neutralizing Collagen Type I solution (3.34 mg/ml rat tail collagen, Corning®, USA) with dropping 1 M NaOH to bring the pH to 7.4. AC hydrogels were prepared by mixing agarose and collagen stock solutions with additional warm DMEM at 37 °C in appropriate volumes to create composite hydrogel with 1.5 mg/ml collagen and 1.5 wt/v% agarose concentrations. Solution was mixed thoroughly and was incubated at 37 °C prior to printing.

Gelatin hydrogel was prepared by dissolving 10 wt/v% gelatin Type A with gel strength 300 (G2500; Sigma-Aldrich, St. Louis, MO, USA) in DMEM by autoclaving the mixture for 15 minutes at 100 ^{o}C. The solution was brought to 37 °C in a water bath.

Characterization: The microspheres were observed under an inverted microscope (Zeiss Axio Vert. A1). The surface morphology of microspheres was viewed under a scanning electron microscope (SEM, JEOL JSM-5600LV) at an accelerating voltage of 5 kV. Cross-section of microspheres was captured under SEM after fracturing the microspheres. The sizes of microspheres, microsphere pores and CLMs were measured using Image J software. Note that

any pore size smaller than micron-scale (<1 μm) were not measurable but they vastly exist in the microspheres. The glass transition (T_g) of the PLGA was determined by differential scanning calorimeter (DSC; TA Instruments, Q200). Hydrolytic degradation of the microspheres was studied in PBS at 37 ^oC. 10 mg of PLGA microspheres were placed in 1 ml of PBS using 1mL centrifuge tube, and incubated at 37 ^oC. Three tubes were centrifuged to remove the supernatant from the microsphere pellet every week, dried under freeze-drier and then weighed and observed under SEM. 1.5 wt/v% agarose, 1.5 mg/ml collagen and the AC composite hydrogel with 1.5 mg/ml collagen and 1.5 wt/v% agarose concentrations were prepared as previously described. The hydrogels were casted on ice, followed by incubation at 37 ^oC for 24 hrs, and then freeze-dried prior to SEM imaging. The hydrogels were imaged at an accelerating voltage of 5 kV.

Mechanical characterization: The uniaxial compressive stress–strain measurements were performed on cylindrical samples after submerging them in DMEM medium (Gibco, UK) supplemented with 10 v/v% fetal bovine serum (FBS; Gibco, UK) and 1 v/v% antibiotic/antimycotic solution (Gibco, UK) at 37 ^oC for three days. Instron 5566 universal testing machine with a load cell of 100 N was used for the testing at a crosshead speed of 1 mm min^{-1}. The cylindrical AC hydrogel, loosely packed PLGA microspheres (~20% v/v) in AC hydrogel and tightly packed PLGA microspheres in AC hydrogel samples (each n=3) were 13–15 mm in diameter and ~4 mm in thickness. The compressive stress (σ) was computed by σ = Load/πr2, where r is the initial unloaded radius; the compressive strain (ε) was defined as the change in the thickness relative to the thickness of the freestanding specimen.

Cell culture: L929 mouse fibroblasts were cultured to access the biocompatibility of the printed construct as a preliminary study. L929 cells were cultured in high glucose DMEM medium

(Gibco, UK) supplemented with 10 v/v% FBS (Gibco, UK) and 1 v/v% antibiotic/antimycotic solution (Gibco, UK).

In order to show that different types of cells can be printed using our methodology, mouse fibroblasts L929, mouse myoblasts C2C12, rat smooth muscle cells A10, rat fibroblasts Rat2, and human epithelial TR146 cells were cultured, seeded and printed. L929, C2C12, and Rat2 cell lines were cultured separately in a high glucose DMEM medium (Gibco, UK) supplemented with 10 v/v% FBS (Gibco, UK) and 1 v/v% antibiotic/antimycotic solution (Gibco, UK). A10 cell line was cultured in a high glucose DMEM medium (Gibco, UK) supplemented with 20 v/v% FBS (Gibco, UK) and 1 v/v% antibiotic/antimycotic solution (Gibco, UK). Meanwhile, TR146 was cultured in HAMS F12 supplemented with 2mM Glutamine (Biological Industries, Israel), 10 v/v% FBS (Gibco, UK) and 1 v/v% antibiotic/antimycotic solution (Gibco, UK). All cells were maintained in a humidified tissue-culture incubator at 37 ^{o}C and with 5% CO_2. L929 and Rat2 were labelled with green fluorescent cell linker (PKH67GL; Sigma–Aldrich, St. Louis, MO, USA); and C2C12, A10 and T146 were labelled with red fluorescent cell linker (PKH26GL; Sigma–Aldrich, St. Louis, MO, USA) according to the manufacturer's instructions prior to cell seeding on microspheres. All the cell lines were seeded separately on the microspheres with the same protocols before printing.

Cell seeding on microcarriers: Prior to cell seeding on microspheres, 0.3 g of microspheres were sterilized by immersing in 70 v/v% EtOH at 4 ^{o}C for 5 hrs. The microspheres were then washed with PBS before suspended in 10 mL of warm (37 °C) cell culture media in a 125 mL siliconized Techne biological stirrer flask (Bibby Scientific Limited, UK). A total cell number of 2.5×10^7 were suspended into the biological stirrer. Pre-warmed (37 °C) cell culture medium was added into the stirrer flask to make a total solution of 40 mL, which the culture is stirred intermittently for 2 min every 30 min at the speed of 30 RPM for the initial cell attachment and

growth. After 6 hrs, the total volume of the culture is increased to 125 mL with warm (37 °C) culture medium and a continuous stirring of 60 RPM is commenced to keep the microspheres in suspension. The stirring was continued for another 20 hrs prior to printing.

Bioink preparation: Preparation of bioinks and the subsequent printing process were conducted in a clean room to ensure a sterile environment for all the transfers. The AC hydrogel and the support material gelatin hydrogel were kept in a water bath at 37 ^{o}C. The microspheres were centrifuged and the supernatant (medium) was removed. The AC hydrogel was quickly added to the microspheres and gently pipetted prior to centrifugation and supernatant (hydrogel) removal. The thin coating of AC hydrogel around the microspheres acts as glue that binds the microspheres together once the temperature of the substrate goes lower than 20 ^{o}C. The bioink (microspheres coated with AC) was kept in water bath at 37 ^{o}C prior to printing.

Constructs printing: Printing of constructs was accomplished using a simple, hand-held printing process using micropipettes (size of 1-10 μL). The micropipette tips were observed under an inverted microscope. An ice platform was prepared to glue the microspheres together. Prior to printing, all the equipment was sterilized by rinsing with 70% EtOH followed by 1 hr of UV bath. Superfrost plus microscope slides (Thermo Scientific, USA) were put onto the ice platform and the bioink was withdrawn into the micropipette. The bioink was then extruded into the desired shape on the glass slides and the 2.5D construct was formed immediately. A 3D construct was built after a layer-by-layer printing. In order to build a tall, complex construct, gelatin hydrogel was utilized as a support material, whereby it is laid down layer by layer to support the biological structure.

Cell viability, proliferation, attachment and immunofluorescence studies: Cell viability of L929 on the microspheres, hydrogel, and printed constructs were accessed using live/dead assay

(Molecular Probes, USA) after 2, 7 and 14 days of culture according to the manufacturer's instructions. An assay solution containing 2 µM calcein-AM and 4µM ethidium homodimer-1 was prepared in Dulbecco's phosphate-buffered saline (DPBS; Gibco, UK). After shifting the samples to new wells and washing the constructs with DPBS, the assay solution was added to the samples incubated at room temperature for 30 min. Fluorescence microscopy (Zeiss Axio Vert. A1) was used to evaluate the live/dead staining of cells in the samples, where the live cells were shown to be green and dead cells were red. The quantification of live cells was computed from the fluorescence readings using the ZEN microscope software. The viability of the cells within the printed 3D constructs were studied using CellTiter-Glo® 3D (Promega, USA). The cells seeded microspheres served as a control. Briefly, after mixing the assay solution with cell culture medium at a 1:1 volume ratio, it was added to each construct with vigorous mixing of well content and incubated for 25 min at room temperature. After incubation, 100 µl of assay solution from each sample was placed into the wells of a white 96-well plate and luminescence intensity was measured using a microplate reader (Ultra Evolution, Tecan). Cell proliferation of the 3D printed constructs was determined by the RealTime-Glo™ MT Cell Viability Assay (Promega, USA), which measures the cell viability in real-time according to the manufacturer's instruction. The cells seeded microspheres were used as a control. Briefly, after mixing the 2× assay solution with cell culture medium at a 1:1 volume ratio, it was added to each construct in a white 96 well plate and incubated at humidified tissue-culture incubator at 37 ^{o}C and with 5% CO_2 for up to 72 hrs. The number of viable cells was determined in culture by measuring the intensity of luminescence signals at several time points from 1 hr up to 72 hrs after culture using a microplate reader.

Cell adhesion and spreading were studied by SEM. Samples were rinsed with PBS and fixed with 2.5% glutaraldehyde in ddH_2O overnight at 4 ^{o}C. Following PBS rinses, the samples were dehydrated through a series of graded alcohol solutions, and then air-dried. Cellular constructs

were gold-coated and observed under the SEM. For immunofluorescence study, simple protocols were used to image the nucleus and F-actin of the constructs. Briefly, NucBlue® Live ReadyProbes® Reagent (Molecular Probes, USA) was utilized to image the nucleus of the constructs by dropping 2 drops of reagent per mL of the cell culture media. The samples were incubated at room temperature for 20 min before imaging. For F-actin immunostaining, the formalin-fixed constructs were permeabilized with Triton X-100 before dropping 2 droplets of ActinGreen™ 488 ReadyProbes® Reagent per mL of DPBS. The samples were incubated at room temperature for 30 min before imaging. All images were acquired with a fluorescent microscope.

Data file S1. Calculations of the initial cell numbers needed before bioprinting of a 1 mm^3 construct.

a

Packing of dry spheres into a known volume was investigated.

Mass of dry spheres per unit volume is 4.08×10^{-4} g/mm^3.

Cells seeding protocol:

3 mg/mL microspheres with 2×10^5 cells/mL.

No. of initial cells/mass of microspheres = 6.67×10^7 cells/g

No. of initial cells/volume = 2.7×10^4 cells/mm^3

<u>Grounds for overestimation:</u>

I) Dry spheres were used in calculation.

II) Before printing, the microspheres were loaded with cells (form microtissues) where the value of microtissues/unit volume will be lower than the calculated value.

b

Assumptions:

I) Cells are spherical with diameter (D) of 20 μm; Spheroids are spherical with D of 200 μm (min) or 600 μm (max).

II) Cells and spheroids have negligible size distributions.

III) Closest spheres packing can be achieved (74% inside the cube)

Consider printing spheres (closely packed) into a cube with volume (V) of 1 mm^2:

I) Cells (assume spherical) loaded in hydrogels

Cells: D = 20 μm

$V_{cell} = 4.19\times10^{-6}$ mm^3

To pack cells inside a cube with $V_{cube} = 1$ mm^3

Closest sphere packing is 0.74.

$V_{\text{sphere could be packed inside the cube}} = 0.74\ mm^3$

No. of cells/volume = $0.74/\ 4.19\times10^{-6}$

$= 1.77\times10^5$ cells/mm^3

Or

No. of cells/volume of hydrogel = 1.77×10^8 cells/ml

II) Tissue spheroids printing in hydrogels

Spheroids: D = 200 μm to 600 μm

$V_{spheroid} = 4.19\times10^{-3}\ mm^3$ to $0.113\ mm^3$

To pack spheroids inside a cube with $V_{cube} = 1\ mm^3$

Closest sphere packing is 0.74.

$V_{\text{spheroids could be packed inside the cube}} = 0.74\ mm^3$

No. of spheroids/volume = $0.74/\ 4.19\times10^{-3}$ to $0.74/\ 0.113$

= 6 to 176

No. of initial cells/Spheroids = 2×10^4 cells/spheroid

No. of initial cells/Volume = 1.2×10^5 cells/mm^3 to 3.5×10^6 cells/mm^3

No. of initial cells/Volume (average) = 1.8×10^6 cells/mm^3

Data file S1. a) Calculation of cells needed to be seeded into microspheres for bioprinting of a 1 mm^3 tissue construct. Overestimation as per reasons stated above. b) Calculations of cell numbers needed for bioprinting of a 1 mm^3 tissue construct for cell-laden hydrogel-based and scaffold-free tissue spheroids-based bioprinting, according to closest packing of spheres inside a volume.

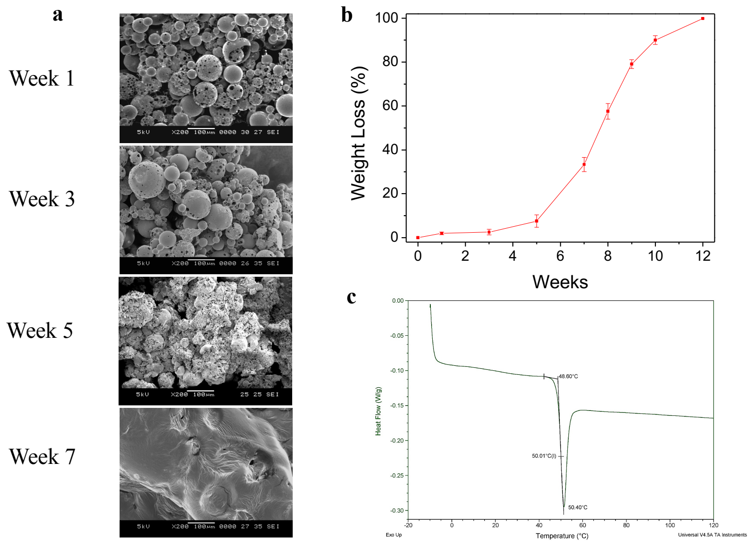

Figure S1. Hydrolytic degradation of PLGA microspheres in PBS at 37 °C. (a) SEM images of PLGA microspheres after 1, 3, 5, and 7 week(s) of hydrolytic degradation in PBS at 37 °C. Microspheres aggregated and the forms of microspheres were lost (not spherical anymore) after 7 weeks. (b) Graph showing the weight loss of microspheres (n=3) with the weeks of hydrolytic degradation. 99% of weight loss occurs after 12 weeks. (c) DSC results showing T_g of PLGA at ~50 °C.

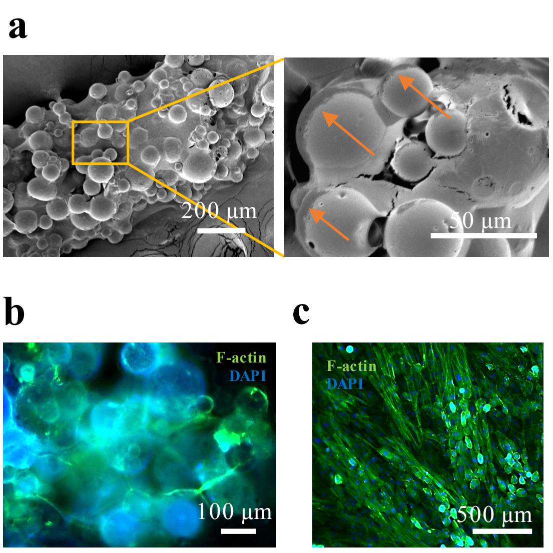

Figure S2. Characterization of printed myoblasts A10 construct. (**a**) SEM images of printed tract using A10 cells. Arrows in the zoomed in image showing the A10 cells found on the construct, with no directional growth observed. (**b-c**) Figures illustrate immunofluorescent staining of a printed A10 cellular construct and A10 culturing on TCPS as control after 3 days of culture.
